# Hormone-Related Cancer and Autoimmune Diseases: A Complex Interplay to be Discovered

**DOI:** 10.3389/fgene.2021.673180

**Published:** 2022-01-17

**Authors:** A Losada-García, SA Cortés-Ramírez, M Cruz-Burgos, M Morales-Pacheco, Carlos D Cruz-Hernández, Vanessa Gonzalez-Covarrubias, Carlos Perez-Plascencia, MA Cerbón, M Rodríguez-Dorantes

**Affiliations:** ^1^ Laboratorio de Oncogenomica Instituto Nacional de Medicina Genomica, Mexico City, Mexico; ^2^ Unidad de Investigación en Reproducción Humana, Instituto Nacional de Perinatología-Facultad de Química, Universidad Nacional Autónoma de México (UNAM), Mexico City, Mexico; ^3^ Unidad de Genómica y Cáncer, Subdirección de Investigación Básica, INCan, SSA and Facultad de Estudios Superiores Iztacala, Universidad Nacional Autónoma de México, Mexico City, Mexico

**Keywords:** autoimmunity, cancer, immune system, sex hormones, inflammation immunotherapy, sex, immunity

## Abstract

Neoplasic transformation is a continuous process that occurs in the body. Even before clinical signs, the immune system is capable of recognizing these aberrant cells and reacting to suppress them. However, transformed cells acquire the ability to evade innate and adaptive immune defenses through the secretion of molecules that inhibit immune effector functions, resulting in tumor progression. Hormones have the ability to modulate the immune system and are involved in the pathogenesis of autoimmune diseases, and cancer. Hormones can control both the innate and adaptive immune systems in men and women. For example androgens reduce immunity through modulating the production of pro-inflammatory and anti-inflammatory mediators. Women are more prone than men to suffer from autoimmune diseases such as systemic lupus erythematosus, psoriasis and others. This is linked to female hormones modulating the immune system. Patients with autoimmune diseases consistently have an increased risk of cancer, either as a result of underlying immune system dysregulation or as a side effect of pharmaceutical treatments. Epidemiological data on cancer incidence emphasize the link between the immune system and cancer. We outline and illustrate the occurrence of hormone-related cancer and its relationship to the immune system or autoimmune diseases in this review. It is obvious that some observations are contentious and require explanation of molecular mechanisms and validation. As a result, future research should clarify the molecular pathways involved, including any causal relationships, in order to eventually allocate information that will aid in the treatment of hormone-sensitive cancer and autoimmune illness.

## Introduction

Cancer does develop in immunocompetent individuals, implying that cancer cells are immune-evading. Even prior to clinical symptoms, the immune system can recognize abnormal cells and work to inhibit their progression, a concept known as cancer immunosurveillance (Burnet, 1957; Thomas, 1982). Increased susceptibility to tumor development in immunocompromised mice, a decrease in those treated with immunostimulatory drugs, and an increase in cancer occurrence in immunocompromised humans receiving immunosuppressive therapy or infected with HIV all support the cancer immunosurveillance theory (Shankaran et al., 2001; Smyth et al., 2005; Grulich et al., 2007). According to the cancer immunoediting concept, the immune system interacts with cancer cells in a three-step process that includes elimination, equilibrium, and escape in which the immune system can either prevent or assist tumor development by interacting with cancer cells (Dunn et al., 2002). During the elimination phase, innate and adaptive immunity collaborate to protect the host by detecting and eliminating cancer cells. This mechanism is favored by cancer cell molecular alterations that result in the expression of modified cell surface markers and patterns referred to as tumor antigens. Some cancer cells can survive the elimination phase and enter a condition of dynamic equilibrium in the second stage. During this stage, the immune system continues to kill tumor cells following a strong immune selective pressure that induces the selection of tumor cells with low immunogenicity, allowing for a greater capacity for survival by evading the immune elimination phase. While these residual cells can remain quiescent in a functional state of lethargy without growing into tumors, they continue to accumulate genetic mutations, a process favored by inflammation (Vinay et al., 2015; Pandya et al., 2016; O’Donnell et al., 2019). These cancer cells enter the escape phase, where they proliferate uncontrollably, demonstrating the immune system’s inability to remove or control altered cells while escaping both innate and adaptive immune defenses. These three steps imply a decrease in immune recognition, an increase in survival, the secretion of molecules that affect effector immune functions, the recruitment of regulatory T cells (Tregs), myeloid-derived suppressor cells (MDSC) and the establishment of an immunosuppressive microenvironment conducive to immunological evasion and tumor progression (Holmgaard et al., 2015; Jang et al., 2017; O’Donnell et al., 2019). These findings, support the notion that evasion of the immune system is one of the hallmarks of cancer (Hanahan and Weinberg 2011).

### Inflammation, Autoimmunity, and Cancer

Inflammation is as an critical determinant of cancer development, this relationship is so compelling that it has been proposed that prescription of anti-inflammatory drugs may reduce the risk of cancer (Retsky et al., 2013; Gilligan et al., 2019). Inflammation occurs as a result of the immune system being stimulated by invading pathogens or endogenous signals such as tissue damage. This response, which aims to restore tissue functionality and homeostasis, is characterized by the identification of immunogenic molecules, changes in vascular permeability, leukocyte recruitment and accumulation, and the release of pro-inflammatory cytokines and chemokines (Medzhitov, 2008). This response results in the elimination of pathogens or stressors, followed by a phase of tissue repair (Barton, 2008; Netea et al., 2017). However, if inflammation persists or the regulatory mechanisms and modulators of the immune response fail, inflammation becomes chronic and may lead to the development of autoimmunity and cancer (Chen et al., 2019; Michels et al., 2021).

**FIGURE 1:**
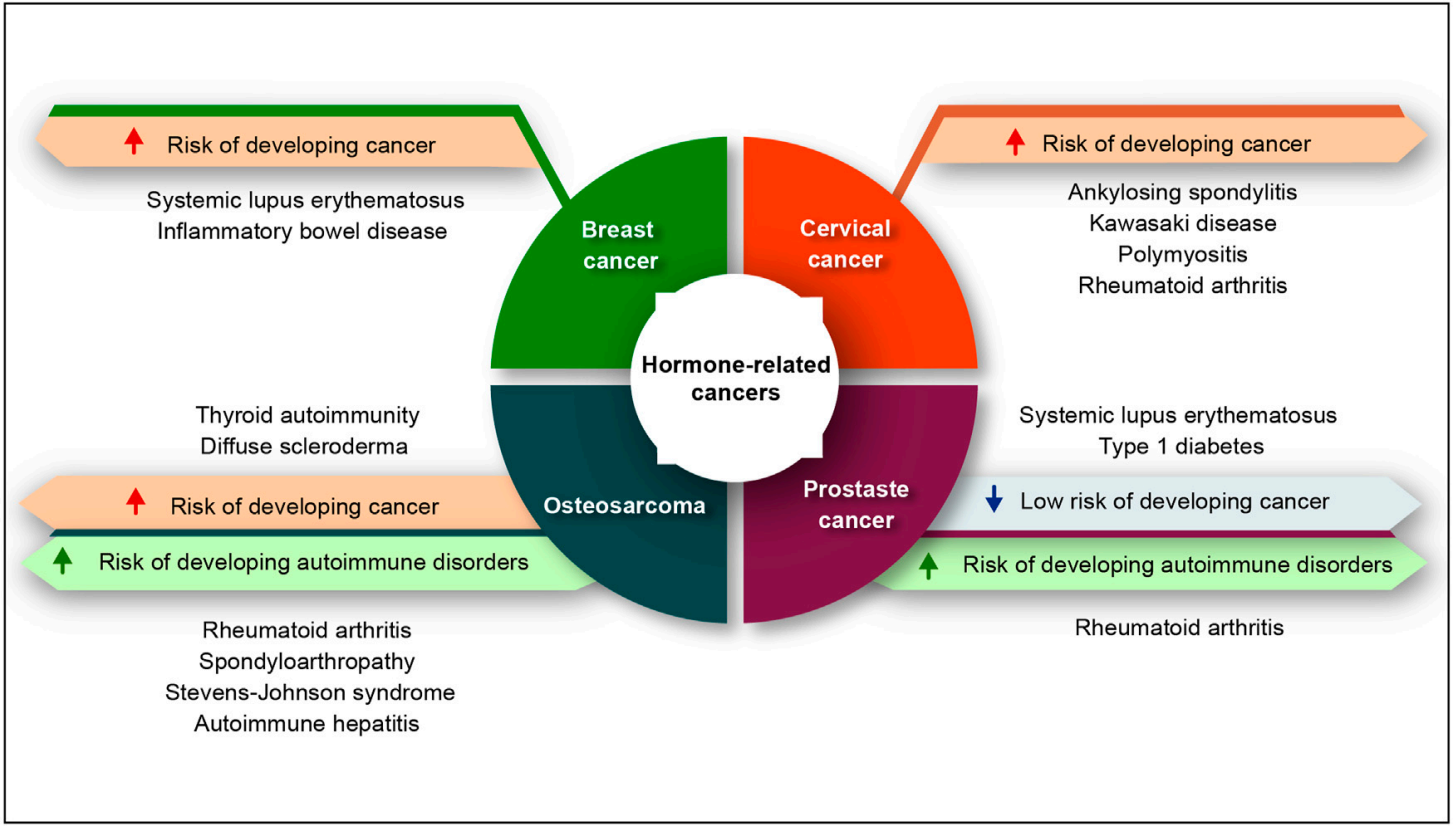
The relationship between autoimmune disorders and hormone-related cancers. The red arrows represent an increased risk of cancer, the green arrows represent an increased chance of autoimmune illness, and the blue arrows represent a lowered risk of cancer.

Virchow proposed the link between inflammation and cancer while examining immune cells within neoplasic tissues, hypothesizing that this immune cell infiltration indicated the development of cancer in areas of chronic inflammation (Virchow and Virchow 1978). Recent investigations have reported a clearer understanding of the link between inflammation and cancer progression. On one hand, neoplasic transformation promotes an inflammatory microenvironment in tumor site and on the other hand and in opposite direction, chronic inflammation increases the risk of cancer development. Both processes converge, resulting in the activation of transcription factors in tumor cells, nuclear factor-κB (NF-κB) and hypoxia-inducible factor 1α (HIF1α), controlling the synthesis of inflammatory mediators such as cytokines and chemokines (Mantovani et al., 2008). Inflammation, regardless of its origin, has a relevant role in the different stages of tumor development, including initiation, promotion, malignant conversion, cell proliferation, angiogenesis, invasion and metastasis (Cai et al., 2019; Ershaid et al., 2019; Liubomirski et al., 2019; Holl et al., 2021). Epidemiological studies report that various forms of cancer malignancies are associated with infection, chronic inflammation, or autoimmunity at one the same tissue (Plummer et al., 2016; Michels et al., 2021). The release of cytokines, interferon-γ (IFN-γ), tumor necrosis factor (TNF), interleukins (IL-1, IL-6), chemokines, prostaglandins, growth factors, and enzymes such as cyclooxygenase and metalloproteinase, as well as the production of reactive oxygen and nitrogen species in a chronically inflamed tissue causes genetic instability and epigenetic alterations, adding to the favorable conditions for the onset of cancer (Fernandes et al., 2015; K.; Nakamura and Smyth 2017). Proliferating tumor cells, tumor stroma cells, including local fibroblasts and macrophages, blood vessels, associated tissue cells, platelets, and infiltrated immune cells generate the pro-inflammatory tumor microenvironment (Allinen et al., 2004; Bindea et al., 2013). In addition, oncogene activation, hypoxia, nutritional deprivation and tumor necrosis already prompted to occur, can lead to the development of an inflammatory environment that collaborates between the autocrine and paracrine glands through cytokines, growth factors, and proteases along with the activation of inflammatory and tumor progression transcription factors such as NF-κB and STAT3 (Pikarsky et al., 2004; Ji et al., 2019). Patients with autoimmune illnesses appear to have a higher risk of cancer as a result of underlying immune system dysregulation or the therapies used to treat these diseases. The relevance of this co-relationship is indicated by epidemiological data on cancer incidence, mortality, and survival from autoimmune disorders. It is possible that autoimmune processes play a role in cancer susceptibility (Figure 1).

Hormones such as androgens, estrogens, progesterone, and thyroid hormones have been implicated in the regulation of immune responses and the development of cancer. Infections, autoimmune diseases, and cancer affect men and women differently. Rheumatic disorders are more common in women than in males, particularly during reproductive life, according to epidemiological evidence (Cutolo et al., 2004). These autoimmune diseases are defined by the presence of autoantibodies and chronic inflammation and include rheumatoid arthritis, lupus erythematosus, psoriasis, celiac disease and inflammatory bowel disease, which have all been associated with the development of cancer (Han, Chen et al., 2015; Simon et al., 2015; Brito-Zerón et al., 2017; Song et al., 2018; Vaengebjerg eta l., 2020; Wan et al., 2021).

## Hormone-Related Cancers and Their Relationship to Autoimmune Diseases

### Prostate Cancer

The role of hormones in cancer development, immune system regulation, and pharmacological response have been widely studied (McEwan and Brinkmann 2000; Özdemir and Dotto 2019; Conteduca et al., 2020). Androgens are responsible for primary and secondary sex characteristics during development. Testosterone is transformed to dihydrotestosterone (DHT), which binds to the androgen receptor (AR), causing conformational changes, nuclear translocation, and gene activation (McKenna and O’Malley 2002). Also, androgens can modulate the immune system by activating the androgen receptor (AR), as well as non-genomic pathways affecting immune cells (Pilo et al., 1981; Simental et al., 1991; Foradori et al., 2008). Nongenomic androgen activity is mediated by androgen binding to plasma membrane receptors, ionic channels, GPRC6A, a class C orphan G protein coupled receptor (GPCR) family member, and ZIP9, a zinc transporter belonging to the ZIP family (SLC39A). Androgens may activate a variety of G proteins, including Gs, Gi, and/or Gq11 (Gn11), as well as extracellular signal-regulated kinase 1 and 2 (ERK1/2), which are involved in a variety of phosphorylation pathways. Erk activity is essential for positive selection in the immune system, and it has an effect on the maturation of CD4 and CD8 T cells, respectively (Fischer et al., 2005; Montaño et al., 2020).

The effect of testosterone on the immune system and cancer cells illustrates the connection between androgens as immune system regulators and cancer development. Prostate cancer (PCa) is one of the most common neoplasms and the second cause of death in industrialized countries (Ferlay et al., 2019). Pca cells overexpress the AR as mechanism of resistance to androgen deprivation therapy (ADT) enhancing recognition by T lymphocytes (Ardiani et al., 2014; Olson et al., 2017). Interleukin IL-6 a pro-inflammatory cytokine overexpressed in the epithelium of prostatic intraepithelial lesions (PIN), may regulate androgen receptor activity promoting cancer development and progression (Hobisch et al., 2000; Lin et al., 2001). Testosterone promotes myelopoiesis in blood cells *via* FOXP3, resulting in the proliferation of immunoregulator cells such as myeloid-derived suppressor cells (MDSCs), Treg, Breg, and M2 macrophages, which sustain immunosuppression (Mierzejewska et al., 2015; Jones and Jørgensen 2020). Testosterone also enhances IL-10 synthesis in T cells and Th2 cytokines while inhibiting Th-1 differentiation (Liva and Voskuhl 2001; Kissick et al., 2014) mice treated with testosterone analogs, showed decreased levels of CD4^+^ CD8 + lymphocytes and increased levels of CD4^+^ CD8- T cells, promoting the pro-inflammatory response and cancer progression (Aboudkhil et al., 1991). An effect of ADT in Pca patients was the reduction in the total level of neutrophils and impaired function after treatment. Also medical castration significantly decreased the amount of CD4^+^CD25 ^+^ T cells, also reduced mitogen-induced CD8^+^ T cell IFN-γ expression, and increased the levels of NK cells while having no effect on the proportion of CD4^+^ to CD8^+^ T cells or the expression of the NK cell-activating receptor NKG2D. These findings imply that androgens can regulate immune cells and may contribute to the maintenance of the physiological balance of autoimmunity by increasing the number of regulatory T cells and the activation of CD8^+^ T cells (Page et al., 2006; Markman et al., 2020). Furthermore, pro-inflammatory molecules have been demonstrated to be overexpressed in men with androgens deficiency (Musabak et al., 2003; Malkin et al., 2004; Bobjer et al., 2013). Androgens can also influence to adaptative immune system. AR is stroma bone marrow expressed and is important for the regulation of androgen-induced B cell development (Olsen et al., 2001). Testosterone promotes the synthesis of TGF- β by the bone marrow stroma while suppressing the production of IL-7, a critical factor in B lymphopoiesis (Tang et al., 1997). Experimental evidence demonstrates in mutant mice AR (-) increased B-cell lymphopoiesis; this study supports the idea that androgens have a direct impact on immune system regulation by decreasing the adaptive immune response (Altuwaijri et al., 2009). Finally, androgens exert their action as suppressors of the immune system. Anti androgen therapies (ADT) used to treat prostate cancer could have a consequence enhancing immune system response, increasing the risk of autoimmune disorders.

Since that up to 90% of lupus patients are female, a protective hormonal component in men appears to be associated with this disease (Rahman and Isenberg 2008). As a result of SLE patients’ decreased testosterone levels, numerous studies associate lupus with a low chance of developing prostate cancer. A meta-analysis of four different cohort studies comparing individuals with SLE to the general population revealed a lowered risk of developing prostate cancer (Bernatsky et al., 2011). Later, similar findings were reported, indicating that SLE patients have a decreased incidence of prostate cancer and melanoma (Song et al., 2018). Rheumatoid arthritis (RA) is a chronic, inflammatory, and systemic disease that begins in tiny joints and spreads to larger joints, as well as other organs such as the eyes, heart, skin, and kidney (Bullock et al., 2018). When rheumatoid arthritis patients were compared to healthy controls, serum testosterone levels were found to be lower and estrogen levels to be higher. 2003 (Tengstrand et al., 2003). Interestingly, therapies for Pca using androgens deprivation therapy (ADT), have been associated with the high risk of being diagnosed with rheumatoid arthritis (RA) (Yang et al., 2018). Type 1 diabetes is another autoimmune condition that is associated with Pca. This disease is caused by pancreatic autoantibodies (Kawasaki, 2014). Numerous meta-analyses and studies have established a link between type 1 diabetes and Pca, and a reduced risk of prostate cancer has been demonstrated, with a 44 percent lower incidence than the general population (Carstensen et al., 2016). This is in contrast to the general trend in type 2 diabetes, which has been associated with a favorable prognosis for prostate cancer (Tseng, 2011). Because prostate cancer tumors exhibit unique genetic and clinical characteristics, it is difficult to predict how their immune microenvironment will respond. However, research in this area is ongoing, and there is still time to investigate the relationship between Pca and autoimmune illnesses. In the future, it will be crucial to unravel this link in order to better understand the molecular mechanisms underlying these disorders and to assess their impact and therapy.

### Osteosarcoma

Osteosarcoma (OS) is the eighth most frequent childhood tumor, occurring at a rate of 2.4 percent. It is more prevalent in adolescents between the ages of 10 and 19, and has been identified as a secondary malignancy in individuals over the age of 65. The incidence of osteosarcoma has also been connected to sex, with men being more afflicted than women, with 5.4 cases per million per year compared to 4 cases per million (Linabery and Ross 2008; Nie and Peng 2018). Long bone metaphyses, such as the femur, tibia, and humerus, are the most common site of osteosarcoma, followed by the long bones of the upper extremities. The lungs are the most frequently expected site of metastasis for this malignancy (Damron et al., 2007). Melatonin (MLT), a circadian rhythm regulator, regulates humoral and cellular immunological responses by limiting T cell proliferation, modifying Treg cell balance, and controlling inflammatory cytokine release. MLT stimulates the differentiation of type 1 regulatory T cells and inhibits the differentiation of Th17 cells. Additionally, melatonin may reduce apoptosis via modulating *BAX* and *BCL* expression, hence avoiding the development of autoimmune diseases (Zhao et al., 2019). In osteosarcoma, for example, melatonin can regulate tumor progression and metastasis. Studies *in vitro* show that melatonin regulates the epithelium-mesenchyme transition (EMT) in the osteosarcoma cell line MG-63 and inhibits the HIF1/Snail/MMP-9 axis suppressing the viability and angiogenesis of cancer cells (Y. Chen et al., 2020). In contrast, melatonin exhibits anti-angiogenic characteristics via miRNAs regulation. Melatonin supplementation of MG-63 and SaOS2 cells can enhance miR-424-5p expression, hence decreasing VEGF levels. This mechanism results in a decrease in the size of blood vessels and the expression of angiogenic factors such as FGF-2 and AGN-1/2, hence inhibiting tumor proliferation and migration (Vimalraj et al., 2020). Another hormone that can influence cancer progression is Leptin which has also been associated with EMT mainly in cancers with hormonal bases such as prostate, ovary and breast (Feng et al., 2016). Adrenomodulin, a vasoactive peptide has a similar structure to calcitonin and can regulate osteosarcoma cell proliferation via the VEGF pathway. Adrenomodulin levels are elevated in osteosarcoma, and it has been hypothesized that it can act as a promoter of angiogenesis and tumor growth in bone (Yao et al., 2019). The growth hormone receptor (GHR) has also been linked to the progression of OS, recent studies show that its expression is higher in OS samples *in vitro*, and its silencing in mouse models reduces migration, invasion, and N-cadherin. This suggests that GHR may act as a promoter of metastatic characteristics in this cancer type (Cheng et al., 2020).

Thyroid hormones (TH) tetraiodothyronine (T4) and triiodothyronine (T3) have a central role in the regulation of systemic energy, growth and basal metabolism, and influence a wide range of organs (Zoeller et al., 2007; Beynon and Pinneri 2016). Thyroid hormones are essential for adults to maintain an optimum level of skeletal growth and bone mass. Thyroid hormone deficiency has an adverse effect on bone development and growth. The TH receptor has been hypothesized to act as a direct negative regulator of bone turnover, acting on both osteoblasts and osteoclasts (Galliford et al., 2005). There is evidence that THs have a direct influence on immune cells such as monocytes, macrophages, lymphocytes, and natural killer cells, modulating immunological responses and inflammation. Hyperthyroidism, defined as an excess in thyroid hormone production or secretion, can be caused by Graves’ disease (GD), toxic multinodular goiter (TMNG) or toxic adenomas (TA), all of which are quintessential cases of thyroid dysfunction (Doubleday and Sippel 2020). GD is defined by a loss of immunological self-tolerance to the TSH receptor (TSH-R), which results in the production of autoantibodies against TSH-R, which stimulate thyroid cells and lead to an increase in the synthesis of thyroid hormones promoting a Th1 immune response and inflammation (Romagnani et al., 2002; Antonelli et al., 2013; Antonelli et al., 2020). Patients with hyperthyroidism have abnormal antibody production, increased reactive oxygen species (ROS) production, and lymphocyte proliferation, whereas those with hypothyroidism have increased pro-inflammatory marker expression and antioxidant capacity, as well as decreased lymphocyte proliferation (De Vito et al., 2011). Specific immune cells, including macrophages, require T3 activity to operate correctly. However, this hormone is synthesized as the prohormone T4. When macrophages capture the T4 hormone, the enzyme deiodinase type 2 (D2) converts it to the active form of T3. D2 function deficiencies and low T3 levels result in a decreased pro-inflammatory response and abnormalities in phagocytosis (Kwakkel et al., 2014). The imbalance in THs production affects immune cell function, enhance the inflammatory responses and increasing the risk of cancer (Y.-K. Chen et al., 2013). Additionally, it has been suggested that the existence of autoimmune illnesses may enhance a patient’s risk of having OS. As with ankylosing spondylitis (AS), a rheumatic illness in which the immune system chronically inflames the spine sacroiliac, and nearby soft tissues, inflammation results in fibrosis and calcification, leading in pain and spinal stiffness (Haslock, 1993). Male AS patients were found to be at an increased risk of developing bone cancer (Chang et al., 2017). Likewise, patients with Kawasaki disease (inflammation of blood vessel walls) and polymyositis (chronic inflammatory myopathy), both autoimmune diseases, have a higher risk of developing bone cancer and other soft tissue sarcomas (Yu et al., 2016). Although additional research is necessary to establish the processes by which autoimmune illnesses can increase the development of osteosarcoma, chronic inflammation in AS is believed to promote the emergence of bone malignancies. We discuss how commonly used cancer-related hormones, like as melatonin and ADM, may operate as critical mediators of OS, as well as the possible significance of ankylosing spondylitis as a risk factor for developing OS. This latter aspect is still controversial, and additional research is needed to establish a definitive connection between the two disorders.

### Breast Cancer

Breast cancer is the leading cause of death and incidence in women worldwide, with rates of 46.3 ASR (age standardized rates) and 13.0 ASR, respectively (Ferlay et al., 2019). Steroid hormones have shown to play a role in the progression of breast cancer at all stages. Early menopause and premenopausal obesity are associated with a reduced risk. However both postmenopausal obesity and menopausal estrogen replacement treatments are associated with a higher risk (Pike et al., 1993). Estrogens are known to regulate a diversity of biochemical processes in males and females, including the nervous, vascular, and skeletal systems (T. Nakamura et al., 2007; Nilsen et al., 2003; Zhu et al., 2002). Estrogens can also influence neutrophil viability, infiltration, chemotaxis, myeloperoxidase production, chemokine and cytokine induction (*IL-6, IL-10, CXCL8*, and *CCL2*), phagocytic activity, and the production of inducible nitric oxide synthase (iNOS) in macrophages, as well as the maturation and differentiation of dendritic cells (DCs) (Khan and Ansar Ahmed 2015). Estrogens can also increase the expression of genes belongin to the immune system such as *CD22*, *SHP-1*, *BCL-2*, and *VCAM-1*, affecting B cell differentiation, survival, activation, and function (Grimaldi et al., 2002). The estrogen receptor (ER) and its coding mRNA are differentially expressed in peripheral blood T cells, B cells, and monocytes, as well as in pre- and postmenopausal females and men’s monocyte populations, implying that estrogens has a significant immunomodulatory effect with well-documented sex dimorphism. This demonstrates their possible significance in the innate and adaptive immune systems, implying that estrogen control may be a treatable target in autoimmune illnesses.

Young women with thyroid disorders are at a higher risk of acquiring primary extra-thyroidal malignancies (Prinzi et al., 2015), and epidemiological evidence suggests a connection between thyroid disorders, whether benign, malignant, or autoimmune, and breast cancer (Fierabracci et al., 2004; Muller et al., 2011; Prinzi et al., 2014). The Hardefeldt meta-analysis confirmed this relationship, finding evidence of an increased risk of breast cancer associated with a diagnosis of goiter or the presence of serum thyroid autoantibodies, particularly anti-TPO and anti-TG (Hardefeldt et al., 2012). Additional research has revealed that the hormone triiodothyronine (T3) can parallel the effects of estradiol on breast cancer cells, stimulating cell proliferation through the induction of growth factors and estrogen pathway proteins (Nogueira and Brentani 1996). Thyroid hormones can also increase estrogen receptor expression and can activate its effect or pathway by binding to the receptor in breast cancer cells (Prinzi et al., 2014). Corresponding signs of thyroid autoimmunity, such as diffuse hypoechogenicity along with the presence of anti-TPOAb and anti-thyroglobulin antibodies, were discovered at higher levels in patients diagnosed with breast cancer in comparison to patients with benign breast disease and healthy controls. The results of this study suggested a strong correlation between thyroid autoimmunity and breast cancer (Giustarini et al., 2006; Jiskra et al., 2007). Diffuse scleroderma is an autoimmune condition that causes regions of the skin to thicken. The skin on the arms, legs, and trunk is more likely to be damaged. Raynaud’s phenomenon (fibrointimal proliferation), extensive fibrosis, and the development of autoantibodies such as anti-Topoisomerase I, anti-centromere anti-polymerase III, anti-U3, and anti-Th/A are characteristics of this disorder. (Liaskos et al., 2017). In 2018, Igusa et al. discovered that patients with anti-pol III autoantibodies had a higher risk of developing breast cancer within the first three years of being diagnosed with diffuse scleroderma when compared to the general population (Igusa et al., 2018), and therefore, adding to the growing evidence connecting breast cancer and autoimmune disorders.

Cyclosporine A (CsA) is an immunosuppressive medication that is used to treat autoimmunity. Recently, this medication was shown to inhibit cancer cell proliferation in the breast cancer cell line MDA-MB-231 by inhibiting the calcineurin (CaN) pathway (Galeazzi et al., 2006; Genere and Stan 2019; Caner et al., 2021). These effects could be beneficial in the management of cancer and autoimmune illness treatments altogether. Aromatase inhibitors (AI) are used to treat estrogen-sensitive breast cancer since they decrease the synthesis of estradiol and estrogen. While estrogens have a natural anti-inflammatory action and may protect against autoimmune illnesses, when estrogen levels are decreased as a result of AI administration, the development of autoimmune disorders may be triggered (Dizdar et al., 2009). Numerous cases of rheumatoid arthritis have been associated to aromatase inhibitor therapies, and other autoimmune diseases such as undifferentiated spondyloarthropathy, Stevens-Johnson syndrome, and autoimmune hepatitis (Tenti et al., 2019). Current evidence support the link between autoimmune diseases and breast cancer, although there are opposing observations (Sarlis et al., 2002) or a lack of validation (Ditsch et al., 2010), there is indeed a complex association between breast cancer and autoimmune disease which should prompt to include more research on the topic to identify the underlying convergent mechanisms of this association.

Progesterone (P4), another female hormone involved in the immune system regulation is required for oocyte maturation, endometrium differentiation, embryo implantation, placental growth, uterine muscle quiescence and mammary gland differentiation (Taraborrelli, 2015; Cable and Grider 2020). Despite its reproductive role, progesterone has been linked to various immune regulatory processes through its action on the innate and adaptive immune system. In the case of monocytic cells, progesterone has been shown to improve monocyte and dendritic cell (DC) differentiation (Ivanova et al., 2005). It has also been demonstrated that progesterone increases IL-15 production via endometrial cell cytotoxicity, which controls uterine NK-cell (uNK cell) expansion (Okada et al., 2000). Progesterone reduce the ability of B cells to present antigen (Zhang et al., 2014), suppress the activity of NK cells (Stopińska-Głuszak et al., 2006) and produce IgG and IgA immunoglobulins in B-cell hybridomas (Canellada, et al, 2002; Lü et al., 2002). P4 can also modulate the cytotoxic activity of decidual lymphocytes because high levels of this hormone suppress the expression of perforin in T cells (Laskarin et al., 1999). It has been proved *in vitro* that P4 shifted naive T cell responses from Th1 to Th2, with increased production of IL-4, IL-5, and IL-10 (Piccinni et al., 1995; Miyaura and Iwata 2002). P4 also induces Treg differentiation and inhibits the Th17 phenotype in human cord blood cells (Lee et al., 2011). During pregnancy, women express membrane progestin receptors (mPRs) and P4 receptor membrane components (PGRMCs) in T cells (Areia et al., 2015). It was shown that P4-induced genes were associated with genes involved with various autoimmune disorders during pregnancy. P4 has been associated with an increased risk of cervical cancer and has been demonstrated to induce the oncogenic transformation of HPV DNA via the RAS oncogene (Pater et al., 1990; Moreno et al., 2002). This gives support to P4’s probable participation in auto immune disease and risks of cancer development.

### Cervix Uterine Cancer

Cervix uterine cancer (CUC) affects women of all ages, with a 13.3 ASR incidence and a mortality rate of 7.3 ASR (Ferlay et al., 2019). Both estrogens and progesterone have been found to influence breast and cervical cancer (Y. Liu et al., 2017). Estrogen and its receptor ER, when combined with human papillomavirus (HPV) oncogenes, promote cervical cancer development (Chung et al., 2010), The most dangerous HPV types are 6, 11, 16, and 18, which account for around 70% of cervical malignancies and 90% of genital warts (Braaten and Laufer 2008). Women with systemic lupus erythematosus have a considerably greater incidence of cervical HPV infection (García-carrasco et al., 2019), which might explain in part this higher CUC risk. In a study of Caucasian individuals with systemic lupus erythematosus (SLE), the occurrences of CUC increased considerably above the expected rates in the general population [SIR 8.15 (95% CI 1.63-23.81)] which could be related to the patient’s autoimmune battle, since there was no proof of other factors that could influence the increased risk (Cibere et al., 2001). Cervical cancer was the most often encountered type of malignancy in a Korean cohort with SLE, although the difference was not statistically significant (Kang et al., 2010). A Danish study of women with a diversity of autoimmune diseases reported that, although there was no increased risk of cervical cancer, there was a higher incidence of this type of cancer in patients who used high doses of azathioprine, an immunosuppressive drug used to treat autoimmune diseases, compared to non-users (Dugué et al., 2015). Furthermore, a meta-analysis identified that individuals with inflammatory bowel disease who use immunosuppressive medications have an elevated risk of high-grade cervical dysplasia and cervical cancer compared to the general population OR = 1.34, 95% CI: 1.23–1.46 (Allegretti et al., 2015). The reports present evidence for several causes of the connection between CUC and autoimmune diseases, such as immunosuppressive treatments associated to elevated cancer risk and the susceptibility of HPV infection in SLE patients that could contribute to a higher incidence of uterine cervix cancer. These studies mainly report associations and correlations between CUC and autoimmune diseases/treatment but follow-up research is needed to confirm this connection and a potential cause-effect relationship.

### Anticancer Immunotherapies

The checkpoint inhibitor treatment, to halt programmed cell death, has been a major lead of cancer immunotherapy. Immunotherapies against cancer have seen a significant increase in discovery and implementation. Anti-cytotoxic T-lymphocyte-associated protein 4 (CTLA-4) and anti-programmed cell death protein 1 (PD-1) antibodies have already been approved for human use, resulting in considerable improvements in clinical outcomes for a variety of malignancies (Seidel et al., 2018). Blocking antibodies against CTLA-4 are particularly effective in prolonging survival and remission rates in cancer patients. CTLA-4 inhibition enhances the likelihood of CD28-mediated T cell activation. However, it raises the risk of developing immune-related adverse events (irAE), which frequently appear as established autoimmune disorders. Patients using CTLA-4 inhibition drugs as ipilimumab are more likely to develop arthritis and other immunological illnesses, including skin, gut, and endocrine-related symptoms (Greisen and Deleuran 2021). CD6 is a transmembrane glycoprotein that is almost exclusively expressed by lymphocytes, including mature T and NK cells. CD6 is a receptor for CD166/activated leukocyte cell adhesion molecule. This interaction contributes to the stabilization of the adhesive connections between T cells and antigen-presenting cells (APCs). CD318 has recently been identified as a second ligand for the CD6 T cell surface glycoprotein. CD318 is widely expressed by cancer cells, and its expression is associated to cancer aggressiveness and metastasis. It has been demonstrated that inhibiting CD6 using an anti-CD6 mAb (UMCD6) significantly increases the ability of human lymphocytes to destroy cancer cells without having negative effects on autoimmune diseases. UMCD6 has a direct effect on the killing ability of CD8^+^ T cells and NK cells. UMCD6 has significant effects on CD4^+^ cell activation and differentiation, which supports the favorable results of its application in animal models of human autoimmune illnesses. These findings suggest that anti-CD6 has distinct effects on CD4^+^ cells that suppress autoimmunity, and direct effects on CD8^+^ and NK cells that promote cancer cell killing. The application of this approach to the treatment of human cancer could prevent the negative autoimmune effects that are typically observed with currently available checkpoint inhibitors (Enyindah-Asonye et al., 2017; Ruth et al., 2021).

## Conclusion

Hormones are critical for immune system regulation. They have the potential to reduce or increase its response, as well as the formation and progression of hormone-sensitive malignancies. Hormones, the immune response, autoimmune illnesses, cancer, and hormonal-based therapies all interact in an intricate biochemical network. Certain autoimmune diseases, such as lupus erythematosus, rheumatoid arthritis, inflammatory bowel disease and thyroid autoimmunity, have been frequently linked to specific forms of cancer or an increased risk of developing them. The immune-cancer interaction has been explained in part by utilizing the fact that the immune system keeps cancer cells “in check” and that hormones not only affect cell growth and progression but also have an effect on the immune system. We discussed various cases of the correlation between hormone-related malignancies and autoimmune illnesses. While some of these findings have not been replicated or validated, and some remain controversial, the hormone-immune-cancer relationships are emerging as an area of research that is contributing to our understanding of the biochemical steps involved in the relationship between the immune system and hormone-related cancers. Future research should describe the molecular mechanisms behind this association, their potential cause-effect relationships, and provide important insights into how to treat hormone-sensitive cancer and autoimmune diseases more effectively.
